# Cultural contexts during a pandemic: a qualitative description of cultural factors that shape protective behaviours in the Chinese-Canadian community

**DOI:** 10.1186/s12889-021-11928-w

**Published:** 2021-10-20

**Authors:** Charlotte T. Lee, Rahim Kanji, Angel H. Wang, Aaida Mamuji, Jack Rozdilsky, Terri Chu

**Affiliations:** 1 Daphne Cockwell School of Nursing, Faculty of Community Services, Ryerson University, 350 Victoria Street, Toronto, Ontario M5B-2K3 Canada; 2 Department of Disaster & Emergency Management, School of Administrative Studies, York University, Toronto, Canada; 3 Department of Communication Studies, Faculty of Liberal Arts & Professional Studies, York University, Toronto, Canada

**Keywords:** COVID-19, Culture, Public health, Collectivism, Chinese, Pandemic preparedness

## Abstract

**Background:**

During the COVID-19 pandemic, there have been significant variations in the level of adoption of public health recommendations across international jurisdictions and between cultural groups. Such variations have contributed to the dissimilar levels of risks associated with this world-changing viral infection and have highlighted the potential role of culture in assigning meaning and importance to personal protective behaviours. The purpose of this study is to describe the cultural factors during the COVID-19 pandemic that shaped protective health behaviours in the Chinese-Canadian community, one of the largest Chinese diasporas outside of Asia.

**Methods:**

A qualitative descriptive design was employed. Content analysis was used to analyze the data from semi-structured virtual interviews conducted with 83 adult Chinese-Canadian participants residing in a metropolitan area in the Province of Ontario, Canada.

**Findings:**

The cultural factors of collectivism, information seeking behaviour, symbolism of masks, and previous experience with severe acute respiratory syndrome (SARS) emerged as themes driving the early adoption of personal protective behaviours within the Chinese-Canadian community during the first wave of COVID-19. These protective behaviours that emerged prior to the first nation-wide lockdown in Canada included physical distancing, mask use, and self-quarantine beyond what was required at the time.

**Conclusion:**

These findings have implications for the development of future public health interventions and campaigns targeting personal protective behaviours in this population and other ethnic minority populations with similar characteristics.

**Supplementary Information:**

The online version contains supplementary material available at 10.1186/s12889-021-11928-w.

## Background

First identified in Wuhan, China, the novel SARS-CoV-2 virus is a highly transmissible pathogen responsible for the 2019 coronavirus disease (COVID-19), an infectious acute respiratory disease that is potentially fatal [1]. The World Health Organization [2] declared COVID-19 a pandemic in March 2020, and owing to its transmissibility and associated morbidity/mortality, lockdowns and protective behaviours have been instituted by various countries in order to restrict the spread of COVID-19. Protective behaviours are defined as actions carried out by the public either independently or due to public health recommendations to control the spread of pathogens, such as mask use and hand hygiene, or avoidant behaviours such as physical distancing and the avoidance of crowds [3]. As Hearne and Nino [4] and Margraf, Brailovskaia and Schneider [5] noted, public health recommendations on protective behaviours have had varying success across international jurisdictions and between cultural groups as their success relies heavily on norms and beliefs that drive an individual’s commitment to enact such behaviours.

Culture, which is defined as the beliefs, attitudes, practices and explanations used to create behavioural norms in a group, can play a key role in the adoption of protective behaviours during the COVID-19 pandemic [6]. Researchers have argued for the role of culture in disease contagion and containment as many factors that are culturally variant can relate to spread, such as styles of greeting (e.g., kisses versus handshakes versus bows), family and household size, and social practices [7]. Psychologists have also postulated that cultural characteristics can influence human behaviour for COVID-19 pandemic responses; for instance, tight cultures with strong social norms and low tolerance for deviance facilitate the enforcement of stringent measures to mitigate transmission of the virus [8]. Additionally, culture can influence the degree to which citizens willingly subsume their individual needs to collective goals, which has significant implications for voluntary compliance with abatement measures during the COVID-19 pandemic, including mask-wearing and social distancing [7].

Cultural differences were noted in previous infectious outbreaks within Chinese diaspora when compared with their mainstream counterparts. For example, Jiang et al. [9] found that during the severe acute respiratory syndrome (SARS) outbreak in 2003, Chinese respondents had a higher risk perception, more anxiety, and implemented more protective behaviours than their non-Chinese counterparts in the Netherlands and the United Kingdom. This difference was hypothesized to stem from value systems, cultural environments, and life attitudes that are unique to Chinese culture [9]. In another study assessing SARS risk perception, Ji et al. [10] found that although Chinese participants estimated a lower perceived risk of infection than Canadian participants, they displayed more protective behaviours to mitigate infection. The authors hypothesized that a possible explanation for this could be the more direct SARS experience Chinese participants had from their social circles [10]. As well, de Zwart et al. [11] noted that though participants from European countries perceived SARS with more severity, Asian participants felt more vulnerable and displayed more self-efficacy in protective behaviours for SARS prevention. This was also hypothesized to be due to the more direct experience with infectious outbreaks and the experience of overcoming epidemics that are both more common in Asian cultural groups [11].

For the present COVID-19 pandemic, there is a paucity of research on the effect of culture on the adoption of protective behaviours. Understanding the factors that affect the adoption of such protective behaviours is imperative during this pandemic as its transmission and the scale of its impact is unprecedented [12]. For context, the SARS epidemic resulted in 8096 cases and a case mortality rate of 9.6% [12]. Contrastingly, even though COVID-19 has a lower case mortality rate, this disease is much more transmissible and at the time of writing, COVID-19 has resulted in 167 million cases and 3.7 million deaths worldwide [2, 12]. Therefore, the aim of this study is to describe the cultural factors that shaped protective behaviours in the Chinese-Canadian community during the COVID-19 pandemic. Since these behaviours are a primary tool in current public health strategies to decrease the spread of COVID-19, it is important to understand culture’s influence to ensure the success of such mitigation strategies.

## Methods

### Design

A qualitative descriptive design was employed to address the research aim. Qualitative description stems from the theoretical framework of naturalistic inquiry, where a truer understanding of a phenomenon in its natural state is thought to occur because it is studied with minimal interference [13, 14]. Additionally, qualitative description offers an analysis that is closer to the data, which yields higher descriptive validity [13]. This is particularly useful in the description of phenomena where little is known due to its use of common terminology understood by researchers and participants [13], thus allowing a rich descriptive summary of the phenomenon of protective behaviour in the context of culture and COVID-19 that can be understood by the general public.

### Setting and participants

Participants were recruited within the Greater Toronto Area (GTA), Canada during the beginning phase of the COVID-19 pandemic in early March 2020. The inclusion criteria were that participants: a) self-identified as ethnically Chinese, b) resided in the GTA for a minimum of one year prior to the onset of COVID-19 (i.e., one year prior to December 2019), and c) did not travel outside of the GTA for more than 3 weeks since December 2019. Maximum variation was used to select key informants within certain “social vulnerability” categories [15] such as varying age groups, gender, sexual orientation, class (via occupation), language and literacy levels, religious or other cultural affiliations, levels of activism, and ethnic identities. Various methods of outreach were also used through the project’s website, television, social media accounts and snowball sampling to increase numbers of participants. This project received ethics approval from the study investigators’ institutions (REB# 2020–125; REB# 2020–083). Informed consent was obtained prior to conducting interviews with the participants and confidentiality was maintained throughout the study.

### Data collection

A one-time semi-structured interview format was used, with an average length of 60 to 90 min per interview. The interviews were primarily conducted in English (64 interviews); however, Mandarin (5 interviews), and/or Cantonese (14 interviews) were also used. Due to pandemic restrictions, interviews were conducted remotely using computer-mediated technology (e.g., Zoom) or by telephone. The interviews were facilitated by trained research assistants or study investigators. All interviews were audio-recorded, transcribed verbatim, and translated into English if necessary. All transcripts were checked for accuracy prior to data analysis and de-identified through the assignment of interview identification numbers.

### Data analysis

Content analysis was conducted to organize transcribed data into a concise summary of key results [16]. Each transcribed interview was coded to determine themes that emerged from the data. The investigators performed an initial coding session across various 10% of the interviews, and through triangulation, arrived at general themes consistent among them. From these themes, key coding categories were assembled. Two teams of coders then used these categories and created additional sub-categories to individually code selected interviews (*n* = 4). Once a consensus was reached on agreed categories and subcategories from these interviews, coders individually coded a second set of interviews (*n* = 4). Coding discrepancies were discussed to establish consensus, ensure consistency and intercoder reliability. From this, a shared coding frame was created.

In addition, back-coding exercises were conducted to reduce unitization issues between coders [17]. The investigators selected 40 sentences from an interview and all coders were instructed to correctly place these sentences in their appropriate coding categories. This process was repeated until an intercoder-reliability percentage of 85 to 90% was obtained. The coding team met after every 10 interviews to reflect on the process and discuss the necessity of additional codes emerging from the data. Meaning units were used (as opposed to predefined blocks of text) in these back-coding exercises. Verification of codes was accomplished by two independent research assistants who examined and extrapolated relevant codes to the research question. Once coding was complete, the next phase of analysis involved an examination of all relevant codes to identify recurring concepts/themes applicable to the research question. This was performed by a team consisting of one outsider and two insiders of the Chinese-Canadian community. The team met bi-weekly to discuss findings and through triangulation arrived upon agreed upon themes for the coded interviews.

### Trustworthiness and rigor

The criteria of credibility, transferability, dependability, and confirmability were used to assess the trustworthiness of the data [18]. Investigator triangulation was used during the coding process to ensure credibility and confirmability. Additionally, the insider and outsider positionality of investigators allowed for an analysis that was framed with cultural contexts because of both insiders’ monopolistic access to cultural knowledge and through cultural detachment due to the outsider’s status [19]. Reflexive journaling was performed by the investigators during content analysis, which also enhanced confirmability. To further enhance credibility, peer debriefing was performed periodically in regard to analysis and findings [20]. To ensure dependability, an audit trail of the research process was maintained through detailed documentation of coding meeting notes, reflexive journals, the recruitment protocol, and all transcribed interviews. Transferability and rigor in the breadth of sampling was achieved through rich descriptive data obtained from interviews with participants from seven ‘social vulnerability categories’ [15], which allowed for a wide range of perspectives within the Chinese-Canadian community. The rigor of this study was also enhanced through plentiful data from 83 different interviews, each lasting 60–90 min, with an average of 20.9 pages of transcribed material per interview. A minimum of 31 questions were asked per interview to elicit a rich knowledge of nuances in experiences and a total of 1735 transcribed pages of interviews were analyzed through line-by-line data analysis over 8 months. Also contributing to rigor were 34 pages of field notes collected within 24 h of each interview to ascertain any implicit (non-verbal) context.

### Findings

#### Participant characteristics

Participants were recruited with a wide range of ages between 18 years and over 65 years. The length of time participants resided in the GTA also displayed significant diversity from one to over 40 years. There was representation from various occupations such as artists, homemakers, and entrepreneurs; however, the majority of participants self-identified as professionals. There were no significant differences between the social vulnerability categories assessed. As noted in Table 1, the majority of participants (70.2%) obtained information about COVID-19 from both mainstream and Chinese news outlets rather than relying on just one cultural form of media.

**Table 1:** Demographic information of interview participants

Variable	Frequency	Percentage
Age (*N* = 83)		
≤ 24 years	13	15.7
25–34 years	10	12.0
35–44 years	22	26.5
45–54 years	16	19.3
55–64 years	9	10.8
≥ 65 years	8	9.6
Unspecified	5	6.0
Length of time residing in GTA (*N* = 83)		
1–10 years	22	26.5
11–20 years	8	9.6
21–30 years	25	30.1
31–40 years	11	13.3
40+ years	12	14.5
Unspecified	5	6.0
Highest Level of Education (*N* = 83)		
High school diploma	9	10.8
College diploma	5	6.0
Bachelor’s degree	38	45.8
Graduate degree	19	22.9
Professional degree	7	8.4
Unspecified	5	6.0
Occupation (*N* = 83)		
Artist	1	1.2
Entrepreneur	5	6.0
Homemaker	4	4.8
International student	3	3.6
Professional	60	72.3
Retired	5	6.0
Student	3	3.6
Unemployed	2	2.4
Sources of Information (*N* = 84)^a^		
Chinese news sources only	5	6.0
Western and local English news sources	10	11.9
Chinese-Canadian news sources	5	6.0
Information from others/expert	2	2.4
Mixed sources (Western, local English & Chinese media)	59	70.2
No news or media exposure	1	1.2
Social media only	2	2.4
Involvement Inside Chinese Community (*N* = 83)		
No	22	26.5
Yes	60	72.3
Unspecified	1	1.2
Involvement Outside Chinese Community (*N* = 83)		
No	36	43.4
Yes	44	53.0
Unspecified	3	3.6

^a^multiple response options allowed

#### Cultural factor 1: collectivism

“One chopstick breaks easily, but 10 chopsticks together are hard to break” (Participant 31).

Collectivism was a strong and consistent theme in the data, with participants voicing the importance of understanding and meeting the needs of not only the Chinese-Canadian community, but the Canadian community at-large. This was represented by one participant who noted: “I think it’s just kind of a respect thing. So, I respect you, you respect me. We all wear a mask. .. Keep each other safe” (Participant 52). Another participant commented on the actions performed for the greater good: “I know some groups raised money to help hospitals buy respirator[s], [and] helped the healthcare workers buy PPE [personal protective equipment]. These actions are good. It shows other people that Chinese [people] care about. .. society” (Participant 64). This desire to protect others was accomplished through self-initiated behaviours such as the early adoption of mask use and voluntary quarantine after international travel at a time when the Canadian government had not yet mandated the use of such measures:

In January to February. .. there was a large community awareness of what was going on and a bit of fear. .. And so, we saw people taking certain protective measures like wearing masks, etc. (Participant 10).

So, I think at that time [February] I started to wear face masks. .. I think they [non-Chinese Canadians] all missed the point that we are wearing mask[s] to protect others, not to protect ourselves.. .. Not many people understand that (Participant 77).

Yeah, we had a couple of kids get pulled out [of school for]. .. a couple of weeks. I think they had just been on vacation in China for the winter break, and so they wanted to do a [voluntary] two-week quarantine. Just for like the safety of their students and safety of the other parents and stuff (Participant 19).

On a broader level, collectivism was demonstrated by participants’ involvement in community initiatives to organize grocery drop-offs for those who were quarantining as well as fundraisers to raise money for PPE for healthcare workers. For example, one participant noted that “[WeChat] groups catering [to] people living in [redacted] and [redacted]. .. [are helping] people who are self-isolating at home, they usually drop off groceries and necessities. These are self-organized groups” (Participant 40). Collectivistic community actions were also demonstrated in the surveillance behaviour amongst Chinese-Canadians. Those who were thought to be subverting social mores during the pandemic were reported on private social media groups:

For example, [when] someone comes back from China, they are immediately posted on WeChat groups. My classmate's neighbour's daughter, she went on a cruise for March break - after she came back, she started to attend a tutoring class. At that time. .. classes were not cancelled. Others reported her in the WeChat group. I have a good friend; she has young children at home. .. [the] young children went to a friend's house to play on a trampoline [and] immediately this was reported by the neighbour.. .. If people think others’ behaviour will affect their life, they [are] exposed. .. on the WeChat group. No one wants to be exposed publicly (Participant 40).

This collectivism in both individual and community actions stems from a sense of social duty or obligation. Specifically, the obligation encompassed a deep personal commitment to the well-being of others. Such a sense of duty was noted by participants to be more pronounced in Chinese culture as compared to mainstream Canadian culture:

I felt it was my duty - our duty to make sure that we don't get other people sick, regardless of what we have (Participant 28).

There are certain kinds of. .. concepts that run stronger [within Chinese culture].. .. I see it as things like filial piety, things like belief in the power [authority]. .. and the control of the state. Senses of obligation and duty (Participant 73).

##### Filial piety as a manifestation of collectivism within the family

Chinese culture recognizes obligations to others; especially the rule of reciprocity in hierarchical familial relationships [21]. Labelled as filial piety, this cultural concept encourages children to place a high priority on the interests and wishes of their parents [21]. Participants described filial piety through reflecting on the needs of older family members and the protective behaviours they engaged in to incorporate these needs during the pandemic:

I have parents who are 90 years old. They used to come down to [participant’s workplace]. .. every day. Now they are home. I won’t let them come out. When I take food to them every two days, I clean everything, make sure there’s no plastic. I put it in the garage. .. They must be away from my eyesight (Participant 3).

##### Collectivism promotes adherence with authorities when trust is developed

Participants emphasized the importance in Chinese culture for following rules and trusting authority. This was emphasized by a participant who stated, “I think in China, people are more conditioned. They are more conditioned to follow the rules by the government because if you don’t, your life won’t be so easy” (Participant 34). However, outside of their jurisdictions of origin, participants challenged local and national public health policies especially regarding the initial dismissal of mask use and a lack of border restrictions. Participants expressed strong wishes for mask use and border restrictions to control the spread of this virus:

Public health officials. .. were saying we don’t need to wear anything. .. Chinese people [were] wear [ing] masks I think in very early days (Participant 61).

I’ll keep wearing the mask, it's obvious. .. I think the biggest thing is the government got to educate people. .. instead of saying don’t wear it (Participant 4).

I don't know why it took so long [to recommend mask use]. It just doesn't even seem logical when they were saying wearing a mask doesn't stop the spread or slow the spread. I don't know what they were thinking (Participant 47).

We have done that petition. .. we called for closing borders way back when. .. if you actually close[d] the border earlier, I don’t think we’ll be in this kind of outbreak now (Participant 49).

These quotes reflected preferences for early public health measures as demonstrated in participants’ homelands because they believed such measures would offer more effective control of viral spread and keep communities safe.

#### Cultural factor 2: information seeking behaviour

##### Information from local and international news sources influenced risk perception

For most participants, pandemic information was gathered from multiple news sources. Most commonly, both Western (including local mainstream media) and Asian news outlets were used in order to understand and appraise the severity of COVID-19, particularly in the early stages of the pandemic when mainstream news did not provide detailed coverage of the outbreaks in Asian countries. Although public health and official government websites were consulted, it is often Chinese and Canadian media outlets that offered first-hand information:

I got the news from SingTao [a Chinese-Canadian newspaper]. .. [but] we have to pay attention [to news in Canada], because we need to live - I have to know the news here. But it is half and half (Participant 53).

I have to admit the first sources I [am] sent to. .. are. .. Canadian-Chinese.. .. I need to double check because I know it might be. .. biased news. So I’ll go for Toronto Public Health. .. ontario.ca.. .. if I have any doubt, I go to those government websites to see what we should do and what’s the next steps (Participant 63).

This pattern of information uptake influenced the adoption of protective behaviours during the beginning stages of the pandemic when mainstream news outlets had little information on COVID-19:

I read news both from here as well as from Hong Kong and other sources. I can understand Chinese. .. so I knew from a very very early stage.. .. That’s the reason why you see a lot of Asians actually start to protect themselves much earlier than people of other Western cultures, right? Like we were wearing masks. .. ahead of what the government is promoting. .. even when they say don’t wear [a] mask, we still wear [a] mask. .. because we know how to protect ourselves and. .. people in our community (Participant 11).

##### Some individuals rely solely on Chinese media

Due to language barriers, participants voiced that some individuals are completely dependent on Chinese sources of information. These sources include news outlets from Chinese speaking jurisdictions in Asia (e.g., China, Taiwan, Hong Kong) and Chinese-Canadian newspapers, which then provide the basis for their assessment of risk and the consequent adoption of protective behaviours. Thus, risk perceptions of the pandemic from these individuals could differ significantly and resulted in drastic, self-imposed public health measures such as avoiding all contacts outside of the immediate household when no community transmission was noted locally.

My grandmother, who starting in December, would not leave the house, and she only watches Chinese news.. .. She was in her condo and she didn’t come out. I don’t even know if she’s gone for like a walk. .. but she’s barely left the house in months. .. She watches Chinese news and so we thought she was overreacting. But maybe not (Participant 83).

Participants then noted a need for official public health information to be translated in order to reach vulnerable community members facing language barriers. One participant stated:

People like my parents who do not speak much English - they are receiving information from Chinese sources, so they do not know much from the Canadian side, and maybe they are just hearing about the numbers and scared, but they are not hearing all the other information. Yeah, so I guess translation is a big thing (Participant 70).

Such information was suggested to be translated and disseminated in partnership with the Chinese community: “I think [public health] can start. .. ethnic-specific alliances. .. and rely on making those partnerships [with the community] to disseminate the information. .. Work with your [community] partners who are choosing to do that as their passion (Participant 73).

##### Use of social media to obtain pandemic information

Participants used social media platforms regularly to obtain health information, mainly via WeChat and WhatsApp. The reliance on social media for information was noted: “70% to 80% of news I consume is from WeChat including translated news” (Participant 40). Such information can be inaccurate and was often acknowledged as being unreliable. However, as one participant indicated, the younger generation of Chinese-Canadians are more likely to appraise and fact-check health information obtained from social media with official government sources as opposed to older generations: “The downside [to WeChat] is that not to generally stereotype, but those of the older generation don’t fact check. .. but they do share a lot through WeChat” (Participant 8).

#### Cultural factor 3: the cultural symbolism of masks

Masks carried a vastly different cultural meaning with participants as compared to the broader Canadian community at the time of the interviews. In Canadian culture, masks are generally a marker of infection or ill health whereas for Chinese-Canadian participants, it is an object that when worn symbolizes practicality, diligence, and personal responsibility:

In Chinese culture. .. people wear masks because they want to protect themselves even when they are not ill. But I think in Western [cultures], when they wear masks that means they are sick (Participant 61).

A Chinese person who’s wearing the mask. .. they must be sick [from a Canadian perspective] and. .. going to infect us, [whereas]. . .a lot of Chinese people just see this as a practical measure, not an anxiety-inducing measure (Participant 8).

I think it’s also interesting because if you wear a mask [in Canada], it signifies that it’s not business as usual. The fact that I’m not seeing so many masks here is almost, perhaps a weird refusal to acknowledge the severity of the situation (Participant 15).

Tied to this culturally specific interpretation of masks was what some described as a resultant emotion of comfort or reduced anxiety when seeing that others had masks on: “The thing is, if I see someone with a mask on, I actually feel more comfortable because I know I’m potentially protected if they have something” (Participant 10). This interpretation of masks is also linked to its ubiquitous use in Asia, whereas one participant highlighted, “the wearing of masks is common. .. in China because of the dust in Beijing from the Gobi Desert. .. you have to wear [a] mask, otherwise you can’t breathe” (Participant 30). Such factors create a different shared meaning for the symbol of a mask in Chinese culture as opposed to Canadian culture.

#### Cultural factor 4: SARS experience

COVID-19 evoked memories of the SARS epidemic for many participants, which triggered anxiety and a sense of urgency. Consequently, a hypervigilance, which manifested as increased protective behaviours at the early stages of the COVID-19 pandemic was apparent. Aside from an emotional trigger, previous experience of SARS resulted in knowledge of protective behaviours (e.g., mask use, physical distancing and quarantine) and how these actions fulfilled the collectivistic notion of protecting self and others. Many participants who endured SARS instinctively adopted protective behaviours early on:

Because the first SARS epidemic started in China and also affected Hong Kong and Toronto, very early on everyone already knew that you should be wearing masks. It is protective. .. Chinese-Canadians do [things] differently from the mainstream and also, they are more nervous (Participant 21).

To some participants, protective actions and public health policies from SARS forged a set of “gold standards” in managing the COVID-19 pandemic. They criticized the authorities when public health messages or policies were not consistent with SARS. One participant emphasized, “On social media there are groups that try to be critical of any public health messages that are not consistent with our SARS experience from 2003.. .. [International travellers] just get the pamphlet and voluntary self-isolation. We did not even have temperature monitoring like most other countries” (Participant 21).

## Discussion

Public health directives that do not adequately account for the beliefs and cultural behavioural patterns of citizens will lack legitimacy and ultimately fail to meet their intended purpose [22]. Huynh [23 , p. 2] emphasized that “human’s behaviours are mainly based on what they perceive others in the community are doing or approve/disapprove of”; thus, culture plays an important role in human behaviour. In the face of the COVID-19 pandemic, it is important to understand cultural factors that influence how the Chinese population behave in order to mitigate the risk of infection, transmission, and disease severity. Our study found four cultural factors influenced protective behaviours of Chinese-Canadians, discussed below.

### Cultural factor 1: collectivism

In collectivistic cultures, interdependency is apparent amongst members within the community [24]. Such interdependency in turn leads to actions for the greater good, or the community’s interests [21, 24]. In particular, collectivism manifests in unified [25] and self-disciplined actions, which are defined as actions motivated by a deep internal discipline to cultivate the self in the vision of humanity, diligence and justice [21]. During the COVID-19 pandemic, many participants in this study adopted protective behaviours early and beyond what was required by the authorities at the time. These self-disciplined actions were driven by an equal motivation for self-protection and the duty to protect others and humanity.

There is recent research to demonstrate that collectivism is useful in promoting protective behaviours. With data collected across 69 countries to examine the relationship between culture and the impact of COVID-19, Maaravi et al. [26] found that the more collectivistic the participants were, the higher the chances they would adhere to COVID-19 prevention measures and health guidelines. Another study by Huang et al. [27] revealed that collectivism can positively predict people’s intention to prevent COVID-19. Similarly, an empirical study conducted by Jiang et al. [24] demonstrated that the cultural distinction of individualism and collectivism had better explanatory power of transmission outcomes of COVID-19 than other factors, including geographical and population factors, religion, and trust. The authors [24] also found that collectivistic societies had much slower spread of COVID-19 in the early stages of the pandemic as they are able to initiate stringent public health measures more quickly and people are more likely to accept governmental controls such as quarantine or staying at home. These results combined with our study findings indicated that the cultural aspect of collectivism is crucial in understanding adherence to prevention measures as well as in developing public health communication strategies. For instance, “mask wearing can protect *you and your family/community* from COVID-19” may be a more effective communication strategy when communicating with the Chinese community as compared to “mask wearing can protect *you* from COVID-19.” This aligns with what Kreuter and McClure [28] asserted, which is that culture is an essential variable in effective health communication.

Additionally, a dimension of collectivistic action is exhibited through filial piety in the findings of this study, or actions that display a respect for family members. As Wills and Morse [29] noted during the SARS epidemic, older Chinese-Canadians respondents strongly adhered to filial piety by taking proactive action to learn how to protect themselves and their family. Participants in our study also exhibited filial piety through bringing food to their elders and limiting their own exposure to the outside world to minimize risk for elderly members of the household. Collectivistic values such as filial piety are important for healthcare professionals to understand in order to provide prevention information that is culturally specific, such as customizing materials for multi-generational households.

### Cultural factor 2: information seeking behaviour

The Canadian experience of SARS has highlighted the importance of clear, comprehensive and timely public health information through a variety of strategies in order to maintain public confidence and decrease anxiety [30]. One such strategy used currently by public health is social media, which allows for bidirectional communication and increases accessibility and access to health information [31]. WeChat, LINE and WhatsApp are the most widely used social media platforms in China, Taiwan and Hong Kong (the most common jurisdictions of origin for Chinese immigrants in Canada [32]) due to characteristics of convenience, promptness and good cross-platform support, thereby enriching and simplifying communication [33]. Our findings suggest that these platforms are also used frequently amongst Chinese-Canadians; therefore, an official public health presence would be beneficial for this community. In fact, Sun and colleagues [33] found that promoting health education through WeChat to be an effective, sustainable and feasible strategy. There is a lack of research regarding if there is a difference in social media use for health information amongst members of the Chinese community as compared to their mainstream counterparts in Canada. Future research addressing this could assess if targeted strategies are needed for the Chinese community rather than a one-size-fits-all approach for all Canadians.

Participants also noted that translation of public health updates was necessary to target vulnerable individuals in the community with language barriers. Currently, the Public Health Agency of Canada and provincial public health bodies have COVID-19 information available in multiple languages [34, 35]. However, early in the pandemic, many community members did not know where this information was or how to find it [36]. Regularly translated statistical information and translated rationales for particular public health measures may be effective in aligning the behaviours of the Chinese-Canadian community with the goals of the broader public health response.

### Cultural factor 3: cultural symbolism of masks

Historically, the meanings attributed to facial coverings have varied significantly between cultures from representing modesty, having cultural significance in particular ceremonies and processions as well as representing protection from infection or pollution [37]. In fact, in some Asian countries, the use of masks parallels folk assumptions of a pure inner self that needs to be protected from the polluted outside world [38]. Such imagery starkly contrasted the view of masks in Western societies early in the pandemic, which resulted in stigma and tension in the Western world [39]. The findings of this study mirror this variance in meaning making as masks symbolized infection and ill health in the broader Canadian culture; however, they symbolized practicality and responsibility with Chinese-Canadian participants. This difference in meaning is partly attributable to the extent of mask use in Chinese culture. In China, as throughout East Asia, mask wearing is a common practice and widely adopted as a practical preventative measure [39, 40].

Similar to those in other Asian countries, people from China wore masks for a variety of reasons prior to the COVID-19 pandemic, including to protect themselves from air pollution, to prevent the spread of the flu, to keep their faces warm in the winter, or to hide their faces when they want to avoid unnecessary social interaction [39]. Mask wearing also became widely popular due to the outbreak of SARS in 2003 and the development of long-term air pollution [39]. In fact, governmental policy makers and the media encouraged the practice as years after SARS when severe smog hit parts of China, official policy and popular opinion reached a consensus on mask wearing [39]. Additionally, during the COVID-19 pandemic, an element of collectivism is also at play as conforming to public concerns for the safety of others over the inconvenience for the self contributed to high mask usage in China prior to it becoming widely accepted internationally [25]. Over time, and particularly accentuated during the COVID-19 pandemic, mask wearing has become a cultural symbol as the ubiquitous image of the masked Chinese citizen in both the Chinese and international media became widespread [40]. Thus, understanding the history and symbolism tied to particular protective behaviours is critical to developing culturally sensitive public health campaigns [41]. For instance, knowledge about the history and underpinnings of masking in East Asian countries may lead to public health measures that diffuse the tension brought forth from the stark contrast of masking.

### Cultural factor 4: SARS experience

Protection Motivation Theory (PMT) describes which factors drive the adoption of particular protective health behaviours in individuals. Originally developed to explain the effect of threat and coping appraisal for the adoption of protective behaviours in the prevention of chronic diseases such as cancer, PMT has since been applied to injury prevention, political issues, and behaviours that protect others [42, 43]. One factor described by PMT that drives protective behaviour is prior experiences with similar disease processes [42]. In our study, it was evident that previous experiences with SARS created a sense of urgency, which ultimately resulted in the early enactment of protective behaviours. Bowman and colleagues [44] corroborated these findings in their cross-sectional study where they found the adoption of protective behaviours to be higher in Hong Kong compared to the United Kingdom as a result of historical exposure to respiratory disease outbreaks such as SARS and high baseline level of risk perception and knowledge. The authors [44] asserted that regional context is important in terms of how people understand risk and how risk drives behaviour, particularly how population sensitization via past infectious disease outbreaks can contribute to protective behaviours. Similarly, Liu [25] noted that the initial vigilance from the Chinese community was in part attributable to previous experience with SARS, with masks in crowded public spaces becoming part of the collectivist norms in China as a result of those epidemics. Such early vigilance in mask use during the current COVID-19 pandemic being propagated by memories of SARS was also noted in our study. Public health officials should account for prior sensitization to infectious disease outbreaks when promoting mitigation strategies for specific communities and in determining how to involve community members in such strategies.

For example, during the SARS epidemic, the Chinese-Canadian community developed a community-led and volunteer-run SARS telephone support line to provide emotional and psychological support to callers, basic knowledge related to SARS, and connected callers to community resources [45]. The community leaders and volunteers recognized that such support needed to account for the different language/cultural barriers within the Chinese community for those from different origins (i.e., Hong Kong, mainland China, and Taiwan [45]). Thus, three separate support lines were created to account for these differences [45]. By recognizing the community’s prior sensitization to SARS, public health officials can not only understand the early vigilance of protective behaviours amongst Chinese-Canadians, but also identify past community initiatives that can give insight into how community empowerment can be incorporated into the emergency and disaster response for future infectious disease outbreaks.

### Strengths and limitations

As described in the participant characteristics, strengths of this study are the large sample size (*N* = 83) for a qualitative design as well as the wide variety of age groups, occupations, jurisdiction of origin (e.g., Hong Kong, mainland China, Taiwanese) and length of time of participants in Canada. Such representation provided a depth of rich, thick qualitative data upon which content analysis was performed. The data was collected through an interview format, and thus a limitation is a risk of recall bias as self-reported data is based on the accuracy of participants’ memories [46]. As well, the interviews were only conducted with Chinese-Canadians in the GTA at a single time in March 2020. Therefore, the perspectives and behaviours of Chinese-Canadians in other Canadian cities, as well as any changes in perspectives and behaviours in our participants as the pandemic progressed are both missing. Additionally, though no significant differences between social vulnerability groups were found, the sample size may not have been adequate enough to detect such differences. Future research should investigate the influence of culture on protective behaviours in Chinese-Canadians in other cities to increase the transferability of findings. As well, research exploring the influence of these cultural factors on protective behaviours within other cultures could also extend the transferability of these results.

## Conclusion

The findings of this study suggest that collectivistic norms, information seeking behaviour, the symbolic meaning of masks, and previous SARS experience contributed to the early enactment of protective behaviours in the Chinese-Canadian community during the COVID-19 pandemic. Therefore, these cultural factors are important for public health officials to consider when initiating and maintaining adherence to public health measures amongst the Chinese-Canadian community. Future public health recommendations should consider the utility of culture in aligning the adoption of specific protective behaviours within targeted communities with public health guidelines.

## Supplementary Information

**Additional file 1.** Standards for Reporting Qualitative Research (SRQR)*.

**Additional file 2.** Interview Guide.

## Data Availability

The datasets used and/or analyzed during the current study are available from the corresponding author on reasonable request.

## Abbreviations

COVID-19: Coronavirus disease of 2019
GTA: Greater Toronto Area
PMT: Protection Motivation Theory
PPE: Personal protective equipment
SARS: Severe acute respiratory syndrome

## References

[1] Beeching NJ, Fletcher TE, Fowler R (2021). Coronavirus disease 2019 (COVID-19).

[2] World Health Organization (2021). WHO coronavirus (COVID) dashboard.

[3] Bish A, Michie S (2010). Demographic and attitudinal determinants of protective behaviours during a pandemic: a review. Br J Health Psychol.

[4] Hearne BN, Nino MD. Understanding how race, ethnicity, and gender shape mask-wearing adherence during the COVID-19 pandemic: evidence from the COVID impact survey. J Racial Ethn Health Disparities. 2020:1–8. 10.1007/s40615-020-00941-1. 10.1007/s40615-020-00941-1 PMC7814861 33469866

[5] Margraf J, Brailovskaia J, Schneider S (2020). Behavioral measures to fight COVID-19: an 8-country study of perceived usefulness, adherence and their predictors. PLoS One.

[6] Singer MK, Dressler W, George S, The NIH Expert Panel (2016). Culture: the missing link in health research. Soc Sci Med.

[7] Mayer JD, Schintler LA, Bledsoe S (2020). Culture, freedom, and the spread of COVID-19: do some societies and political systems have national anti-bodies?. World Med Health Policy.

[8] Chen SX, Lam BC, Liu JH, Choi HS, Kashima E, Bernardo AB (2021). Effects of containment and closure policies on controlling the COVID-19 pandemic in East Asia. Asian J Soc Psychol.

[9] Jiang X, Elam G, Yuen C, Voeten H, de Zwart O, Veldhuijzen I, Brug J (2009). The perceived threat of SARS and its impact on precautionary actions and adverse consequences: a qualitative study among Chinese communities in the United Kingdom and the Netherlands. Int J Behav Med.

[10] Ji L, Zhang Z, Usborne E, Guan Y (2004). Optimism across cultures: in response to the severe acute respiratory syndrome outbreak. Asian J Soc Psychol.

[11] de Zwart O, Veldhuijzen IK, Elam G, Aro AR, Abraham T, Bishop GD, Voeten H, Richardus JH, Brug J (2009). Perceived threat, risk perception, and efficacy beliefs related to SARS and other (emerging) infectious diseases: results of an international survey. Int J Behav Med.

[12] Fung TS, Liu DX (2021). Similarities and dissimilarities of COVID-19 and other coronavirus diseases. Annu Rev Microbiol.

[13] Sandelowski M (2000). Whatever happened to qualitative description?. Res Nurs Health.

[14] Colorafi KJ, Evans B (2016). Qualitative descriptive methods in health science research. HERD Health Environ Res Des J.

[15] Thomas DSK, Phillips BD, Lovekamp WE, Fothergill A (2013). Social vulnerability to disasters.

[16] Elo S, Kyngas H (2008). The qualitative content analysis process. J Adv Nurs.

[17] Campbell JL, Quincy C, Osserman J, Pedersen OK (2013). Coding in-depth semistructured interviews: problems of unitization and intercoder reliability and agreement. Sociol Methods Res.

[18] Lincoln YS, Guba EG (1985). Naturalistic inquiry.

[19] Merton RK (1972). Insiders and outsiders: a chapter in the sociology of knowledge. Am J Sociol.

[20] Woo K (2019). Trustworthiness and integrity in qualitative research. Polit & Beck Canadian essentials of nursing research.

[21] Wang G, Liu Z (2010). What collective? Collectivism and relationalism from a Chinese perspective. Chinese J Commun.

[22] Kasdan DO, Campbell JW (2020). Dataveillant collectivism and the coronavirus in Korea: values, biases, and socio-cultural foundations of containment efforts. Adm Theory Prax.

[23] Huynh TLD (2020). Does culture matter social distancing under the COVID-19 pandemic?. Saf Sci.

[24] Jiang S, Wei Q, Zhang L (2021). Individualism vs. collectivism and the early-stage transmission of COVID-19.

[25] Liu JH (2021). Majority world successes and European and American failure to contain COVID-19: cultural collectivism and global leadership. Asian J Soc Psychol.

[26] Maaravi Y, Levy A, Gur T, Confino D, Segal S (2021). “The tragedy of the commons:” how individualism and collectivism affected the spread of the COVID-19 pandemic. Front Public Health.

[27] Huang F, Ding H, Liu Z, Wu P, Zhu M, Li A, Zhu T (2020). How fear and collectivism influence public’s preventive intention towards COVID-19 infection: a study based on big data from the social media. BMC Public Health.

[28] Kreuter MW, McClure SM (2004). The role of culture in health communication. Annu Rev Public Health.

[29] Wills BS, Morse JM (2007). Responses of Chinese elderly to the threat of severe acute respiratory syndrome (SARS) in a Canadian community. Public Health Nurs.

[30] Kothari A, Foisey L, Donelle L, Bauer M (2021). How do Canadian public health agencies respond to the COVID-19 emergency using social media: a protocol for a case study using content and sentiment analysis. BMJ..

[31] Moorhead SA, Hazlett DE, Harrison L, Carroll JK, Irwin A, Hoving C (2013). A new dimension of health care: systematic review of the uses, benefits, and limitations of social media for health communication. J Med Internet Res.

[32] Statistics Canada (2007). The Chinese community in Canada.

[33] Sun M, Yang L, Chen W, Luo H, Zheng K, Zhang Y, Lian T, Yang Y, Ni J (2020). Current status of official WeChat accounts for public health education. J Public Health.

[34] Government of Canada. Coronavirus disease (COVID-19): awareness resources. 2021. https://www.canada.ca/en/public-health/services/diseases/2019-novel-coronavirus-infection/awareness-resources.html. Accessed 19 May 2021.

[35] Ontario. COVID-19 communication resources. 2021. https://www.ontario.ca/page/covid-19-communication-resources. Accessed 19 May 2021.

[36] Owen B (2020). Community groups filling gaps in translation of COVID-19 information.

[37] van der Westhuizen H, Kotze K, Tonkin-Crine S, Gobat N, Greenhalgh T (2020). Face coverings from COVID-19: from medical intervention to social practice. BMJ..

[38] Burgess A, Horii M (2012). Risk, ritual and health responsibilisation: Japan’s ‘safety blanket’ of surgical face mask-wearing. Sociol Health Illn.

[39] Ma Y, Zhan N. To mask or not to mask amid the COVID-19 pandemic: how Chinese students in America experience and cope with stigma. Chin Sociol Rev. 2020:1–26. 10.1080/21620555.2020.1833712.

[40] Sin MSY (2016). Masking fears: SARS and the politics of public health in China. Crit Public Health.

[41] Crawford J, Ahmad F, Beaton D, Bierman AS (2015). Cancer screening behaviours among south Asian immigrants in the UK, US, and Canada: a scoping study. Health Soc Care Community.

[42] Floyd DL, Prentice-Dunn S, Rogers RW (2000). A meta-analysis of research on protection motivation theory. J Appl Soc Psychol.

[43] Rogers RW (1975). A protection motivation theory of fear appeals and attitude change. J Psychol.

[44] Bowman L, Kwok KO, Redd R, Yi Y, Ward H, Wei WI (2020). Public perceptions and preventive behaviours during the early phase of the COVID-19 pandemic: a comparative study between Hong Kong and the United Kingdom. J Med Internet Res.

[45] Dong W (2008). Beyond SARS: ethnic community organization’s role in public health – a Toronto experience. Promot Educ.

[46] Hassan E (2006). Recall bias can be a threat to retrospective and prospective research designs. Int J Epidemiol.

