# Supplementary material for: Cultural contexts during a pandemic: a qualitative description of cultural factors that shape protective behaviours in the Chinese-Canadian community
Source: BMC Public Health. 2021 Oct 20;21:1897. doi: 10.1186/s12889-021-11928-w (PMC8526107; doi:10.1186/s12889-021-11928-w)
Supplement: Supplementary file 2 — Additional file 2. Interview Guide. [file 12889_2021_11928_MOESM2_ESM.docx]

**Interview Guide:**

**Demographic Details**

- Name and area of residence?
- Age group: <24 years, 25 - 34, 35 – 44, 45 – 54, 55 – 64, 65 – 74, >75 years
- What ethnicity do you identify with?
- What languages do you speak?
- Highest education received?
- Occupation?
- How long have you been in the Greater Toronto Area? Canada?

**Community Engagement**

- Are you involved with organizations/community groups?
  - a) within the Chinese community?
    - ***Follow up:*** What is the cultural make-up of your network?
  - b) broader, non-Chinese community?

**Before COVID-19**

- How would you describe the relationship amongst people of different Chinese backgrounds or heritages residing in the GTA (Taiwan, China, Hong Kong)?
- How well integrated do you feel the Chinese community is with the broader/mainstream Canadian community prior to COVID-19?

**COVID-19 Observations: The Start**

- Where do you get your information or news?
  - - ***Follow Up***: How reliable/credible do you think that information is?
- When did you start paying attention to COVID-19? What were your thoughts about it when it first began? What are you feeling now?
- Did you or anyone in your Chinese community take actions to raise awareness about the risks early on?
- Do you think different Chinese groups perceive risks and/or react to COVID-19 differently?

**COVID-19 Observations: Impacts**

- What challenges/impacts due to COVID-19 have you experienced personally?
- What challenges/impacts due to COVID-19 have you observed amongst other members of the Chinese community in the GTA?
- Have you experienced treatment that is different since COVID-19?
- Did you decide to wear a mask/when? If not, why?
  - ***Follow Up:*** Did you want to wear a mask but feel pressure not to wear it?
- Have you observed other members of the Chinese community in the GTA being treated differently since COVID-19?
- Have you observed discrimination from within the Chinese community (i.e. between individuals from Taiwan/Hong Kong/mainland China)?
- Have you observed those outside the Chinese community being treated differently since COVID-19? (e.g. people from Iran, Italy, Spain, etc. when COVID-19 numbers surged there?)

**COVID-19 Observations: Coping Capacity (current state)**

- Where do you seek support during this challenging time?
- What are you and your cultural community doing to overcome some of the challenges that you are facing following COVID-19?
- How can emergency managers/public health officials/social services enhance the capacity of the Chinese community/accommodate the needs of the Chinese community during infectious diseases?
- What are you and your cultural community doing to overcome some of the challenges as they relate to being treated differently since the outbreak?
- Are there things that you/others in the Chinese community doing to help the broader society during COVID-19? If yes, how do the people you are helping respond?

**Combating Stigma (forward looking)**

- What else do you think ethnic Chinese people can do to help themselves to effectively address the stigma within the Chinese community since COVID-19?
- What can those outside of the Chinese community do to address/combat the stigma that the Chinese community is currently experiencing, i.e. to be effective allies?

**Closing Questions:**

- We would like to meet with members of the general Chinese community in the GTA. Is there anyone in the Chinese community that you think we should aim to reach out to who can share a useful perspective?
- Is there anything you would like to add?
